# Ethnobotanical Inventory of Plants Used by Mountainous Rural Communities in NW Portugal

**DOI:** 10.3390/plants13192824

**Published:** 2024-10-09

**Authors:** Alexandre Sá, Teresa Letra Mateus, Nuno V. Brito, Cristiana Vieira, Ângela M. Ribeiro

**Affiliations:** 1 Technological Center for AgriFood Sustainability (NUTRIR), CISAS—Center for Research and Development in Agrifood Systems and Sustainability, Polytechnic Institute of Viana do Castelo, Monte de Prado, 4960-320 Melgaço, Portugal; alexandre.sa@ipvc.pt (A.S.); tlmateus@esa.ipvc.pt (T.L.M.); nunobrito@esa.ipvc.pt (N.V.B.); 2 Veterinary and Animal Research Centre (CECAV), UTAD—Universidade de Trás-os-Montes e alto Douro, Associate Laboratory for Animal and Veterinary Sciences (AL4AnimalS), Quinta de Prados, 5000-801 Vila Real, Portugal; 3 Instituto de Saúde Pública da Universidade do Porto (EpiUnit), Laboratório Para a Investigação Integrativa e Translacional em Saúde Populacional (ITR), Universidade do Porto, Rua das Taipas, nº 135, 4050-091 Porto, Portugal; 4 One Health Toxicology Research Unit (1H-TOXRUN), University Institute of Health Sciences, CESPU, CRL, 4585-116 Gandra, Portugal; 5 Museu de História Natural e da Ciência da Universidade do Porto (MHNC-UP/Porto/PRISC), Praça Gomes Teixeira, 4099-002 Porto, Portugal; cvieira@mhnc.up.pt

**Keywords:** Alto Minho, Portugal, NW Iberia Peninsula, local knowledge systems, utilitarian plants, ethnobotany

## Abstract

Mountains matter. Rural subsistence communities living in areas with high biodiversity, such as mountains, are hotspots of ecological knowledge. However, modern lifestyles may threaten this unique cultural heritage. Our study aimed to document and analyze information on plants used to fulfill the everyday needs of the people in three rural communities in NW Portugal. Fieldwork was carried out for a period of one year and information was collected through face-to-face semi-structured interviews. A total of 98 species, belonging to 46 families, were identified, and 142 vernacular names were recorded. Ethnobotanical richness was similar among the studied communities. The five most frequently cited species were: *Pterospartum tridentatum*, *Erica arborea*, *Ruta graveolens*, *Zea mays* and *Chamaemelum nobile*. Phanerophytes and hemicryptophytes comprise nearly 81% of the list. The top three uses categories (total 14) were: medicine, fuel and ritual. Digestive, skin and respiratory symptoms were the most often conditions treated with plants. Medicinal plants were used fresh and dried, mostly as infusions. The insights gathered here are important for the preservation of the cultural heritage of the local communities. Moreover, the data are of considerable scientific interest because it provides the fundaments for future studies that aim to validate/invalidate specific uses.

## 1. Introduction

Ecological knowledge plays a crucial role in understanding the interactions between humans and the natural world. However, there has been an increasing separation between people and nature [1,2], which, in turn, contributes to the loss of local ecological knowledge. It is acknowledged that modern lifestyle has shaped the degree and depth of knowledge about wildlife. Factors such as the systems of formal education implemented at national levels, migration, land use change and urbanization are associated with the loss of the ability to recognize, name and use biological resources [3,4,5]. The cumulative body of knowledge, practices and beliefs, evolving by adaptive processes and passed through generations by cultural transmission, is known as local traditional knowledge [6] and includes ecological knowledge. This may include information such as the harvest period of a particular resource, the best storage methods and harvesting tools, and complex belief systems, all fundamental for people’s livelihood and health. The value of local knowledge is internationally recognized as a way of protecting and ensuring the sustainable use of ecosystems and a source of information related to resilience and adaptation to the environment. In 2003, UNESCO deemed local ecological knowledge worthy of protection through the Convention for the Safeguarding of the Intangible Cultural Heritage [7] and further recognized “that respect for Indigenous knowledge, cultures and traditional practices contributes to sustainable and equitable development and proper management of the environment” in the UN Declaration on the Rights of Indigenous Peoples [8].

In recent decades, the ecological knowledge of rural communities has become the object of scientific studies [6,9], with the argument that it is fundamental to ensure sustainable socio-ecological systems in high biodiversity areas [10,11,12]. This is particularly relevant in mountain socio-ecological systems (MSESs) because the ecosystem services they provide depend largely on land use shaped by the long-term interaction of humans with nature [13]. While MSES are ecological knowledge hotspots due to their high botanical diversity and cultural diversity [14], they are also regarded as highly vulnerable. Mountain rural communities are currently facing unprecedented rates of societal and environmental changes, namely a decline in local knowledge heritage [5,15,16,17] and the impacts of climate change on biodiversity [18].

In Portugal, MSESs are also iconic and host economic activities relevant at the national and regional levels. Concomitantly, most mountains (18% country’s territory) are classified as protected areas (national or international legal protection) for their high biological diversity and because they host unique cultural heritages bound to nature, particularly plants, which have always been a crucial resource. Despite all these, the documentation of local knowledge associated with plants is still scarce. The beginning of the country’s systematic ethnobotanical inventories (to address the relationships between humans and plants) in MSESs was sponsored by the national conservation authority (Instituto para Conservação da Natureza—ICN) in the late 1990s. Since then, and despite the increasing interest and efforts towards documentation of plant uses (e.g., Refs. [19,20,21,22]), to the best of our knowledge, no study has been devoted to the peculiar socio-ecological systems in NW Portugal, where livelihoods are based on agro-silvo-pastoralism in areas with high biological diversity (e.g., Peneda-Gerês National Park and Natura 2000 Special Area of Conservation Serra d’Arga) at the southern edge of the Atlantic biogeographical region.

To address this knowledge gap, we set out to document and evaluate the diversity of knowledge associated with plants—ethnobotanical inventory—in the rural communities of NW mountains of Portugal, with the rationale of contributing to biocultural heritage preservation in areas relevant to biodiversity conservation. The aims of this study were as follows: (i) to record the ethnobotanical knowledge of the MSES of Alto Minho; (ii) to highlight local plant resources of particular interest for medicinal purposes and (iii) to provide knowledge that can support sustainable economic activities in communities that are currently struggling with depopulation. Systematic ethnobotanical studies are of paramount importance to assess and mitigate the potential loss of valuable local knowledge and practices in areas that are succumbing to rural abandonment, changes in land use and the breakdown in transmission to younger generations.

## 2. Results

### 2.1. Diversity of Utilitarian Plants

We interviewed forty-four informants (thirty-eight females and seven males) whose ages ranged from 50 to 94 years, with seventeen from Castro Laboreiro, sixteen from Serra d’Arga and eleven from Soajo. Our study revealed that 106 plant species and two lichens (two genera from two families) had utilitarian purposes. From all botanical references, one citation was for Bryophyta (general designation used) and eight were for Tracheophyta, for which we could not ascertain the species; hence, 98 were plants identified to the species level (Supplementary Materials Table S1).

Across all informants, we collected 641 use reports for the 98 vascular plant species: 257, 189 and 190 reports for Castro Laboreiro, Serra d’Arga and Soajo, respectively. The mean informant report was 14 citations ± 10 (range 1–44). We found no significant effect of locality on the number of citations per species (Kruskal–Wallis Chi-squared = 1.001, df = 2, *p* = 0.606). Twenty-three species were used in the three communities and thirty were shared between pairs (Supplementary Materials Figure S1).

Given the above information, we proceeded with the analyses, lumping the three mountain communities and accounting exclusively for the plants to which species-level identification was unambiguous (97 species).

#### 2.1.1. Use Categories

**Figure 1:**
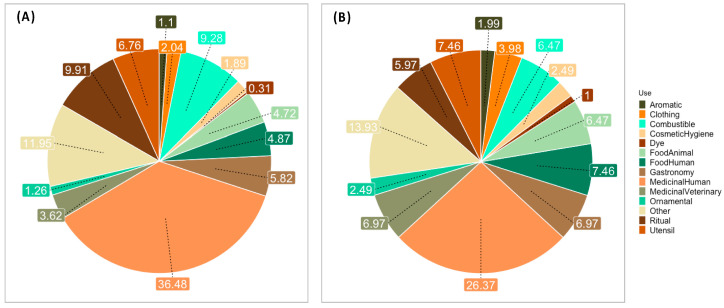
Uses for all plants mentioned and identified to the species level (98 species) as categorized into 14 classes. (**A**) Proportion of reports per category. (**B**) Proportion of species per category. Medicinal purpose was the most frequently cited (36.48%, 232 mentions) and the most species-diverse category (26.37%, 53 species).

A total of 14 use categories were defined and the use category with most references (*n* = 220) was human medicine, followed by ritual (*n* = 63) and fuel (*n* = 59) (Figure 1A). In terms of plant species, these correspond to 53, 12 and 13 species, respectively. The category with fewer mentions (*n* = 2) was dye, with two species identified. The five most frequently cited species were *Pterospartum tridentatum* (L.) Willk. (45 use reports), *Erica arborea* L. (27 use reports), *Ruta graveolens* L. (23 use reports), *Zea mays* L. (23 use reports) and *Chamaemelum nobile* L. (20 use reports). *P. tridentatum* was mentioned as medicine and fuel, twenty and six times, respectively. Maize is the most often reported plant within the human food category; yet it was also used for medicinal purposes (five reports). Tree heather (*E. arborea*) was mainly used as fuel either for cooking or house heating, and rue (*R. graveolens*) was used in ritual practices.

#### 2.1.2. Plant Families, Origin and Life Form

**Figure 2:**
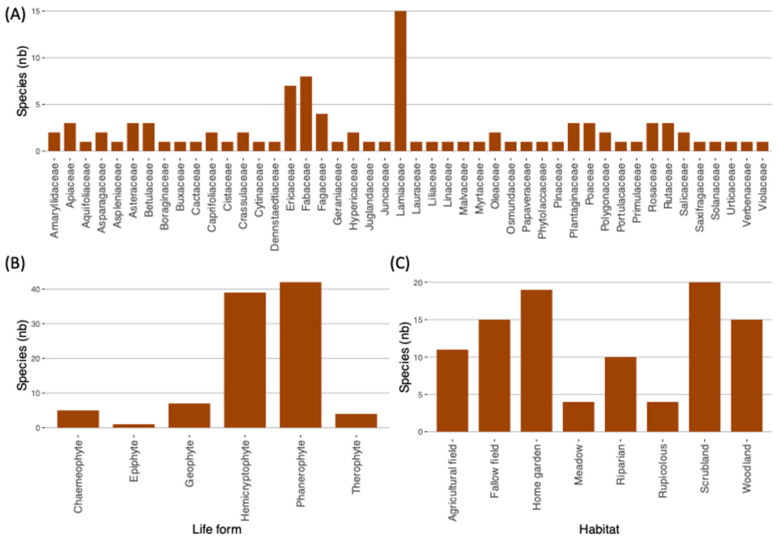
Details about the ethnobotanical resources exploited by three studies communities of NW Portugal: (**A**) plant families, (**B**) life form and (**C**) broad habitat groups where harvested.

We identified 47 families, all vascular plants, and two lichens (Umbilicariaceae and Parmeliaceae). However, discarding the taxa in which a species-level identification could not be achieved, we were left with 46 families of vascular plants (Figure 2A). Lamiaceae was the most frequent with fifteen species, followed by Fabaceae and Ericaceae with nine and eight species, respectively. 

According to the species life form (sensu Raunkiær; see Materials and Methods for details), the mentioned vascular plants were as follows: forty-two phanerophytes (trees and shrubs), thirty-nine hemicryptophytes (die back each year), seven geophytes (with underground storage organs), five chamaephytes (small shrubs and herbs), four therophytes (annuals), and one epiphyte (Figure 2B and Table S1). Nearly 70% of the species used were sourced from the wild (Figure 2C): mainly, scrubland *(n* = 20), woodland *(n* = 15) and riparian *(n* = 10). The remaining ethnobotanical resources (30%) were assigned to anthropogenic environments, such as agricultural fields and home gardens (Figure 2C and Supplementary Materials Table S1).

#### 2.1.3. Utilized Parts

We collected information on 16 parts of plants used for different purposes: trunk, stem, leaf, flower, stigma, seed, fruit, peduncle, root, rhizome, tuber, bulb, latex, sap, bark and gall. Shoots, i.e., leaves and stems, were the most frequently mentioned plant part (39%); leaves and flowers accounted for 18% and 8%, respectively. Yet, it was frequent to use stems with leaves and flowers together (2%). The less commonly used parts were peduncle and sap (0.15%; one use report each).

### 2.2. Quantitative Analysis of Useful Plants: Ethnobotanical Indices

The number of different plant species used by the communities, i.e., ethnobotanical richness (R) was 98. The relative frequency of citation (RFC) of the reported species ranged from 0.02 to 0.55, with *P. tridentatum*, *E. arborea*, *R. graveolens*, *Laurus nobilis* L. and *Secale cereale* L. being the top five taxa with the highest RFC. The cultural importance index ranged from 0.02 to 1.05, and the most culturally significant species were, by descending order, *P. tridentatum*, *S. cereale*, *Zea mays*, *E. arborea* and *R. graveolens*. *P. tridentatum* was mainly used as medicine, but was also mentioned as relevant for fuel, condiment and ornamental. Rye, *Secale cereale*, was grown for human (seed) and animal food (straw) and for thatching roofs (straw). *Erica arborea* was chopped for use as a combustible, animal food and for utensils craft (brooms for cleaning the wood-fired oven used for bread baking). Rue, *Ruta graveolens*, was kept in home gardens for its magical purposes, but there were also mentions of medicinal uses as abortive and painkiller. Laurel, *L. nobilis*, like rue, was used in religious ceremonies—blessed on Palm Sunday—in addition to its main use as an aromatic and fuel. Regarding the informant consensus factor (ICF), the highest values were recorded for ritual (ICF = 0.82), followed by fuel (ICF = 0.79) and human medicine plants (ICF = 0.77) (Table 1).

We found a total of 142 vernacular names to designate the 98 species. Nearly 36% of the species had more than one common name, and the phytonym linguistic diversity index was 1.50 (mean number of vernacular names per species). Frequently known in the communities by two names were twenty-two species; thirteen had three local designations and one species had as much as five vernacular names. Overall, the botanical linguistic richness (BLR) was 1.44. The estimation per locality was similar: Castro Laboreiro = 1.20, Serra d’Arga = 1.16 and Soajo = 1.12. BLR values larger than one (1) indicate that taxonomic entities have multiple vernacular designation, which expresses the linguistic richness of the community.

### 2.3. Local Medicinal Species

This was the largest class of use with sixty-three plants: fifty-three species (thirty-two families) used to treat humans and fourteen for animal afflictions (nine families), with five serving both purposes. Lamiaceae was the family with more species (*n* = 10) followed by Apiaceae, Asteraceae and Rutaceae (*n* = 3). Ninety-two percent of the plants used for human medicine purposes are perennials that keep their growth point above the surface of the ground during the unfavorable season (twenty-seven hemicrytophytes, sixteen phanerophytes and five chamaephytes). Commonly used plant parts were aerial parts (shoot and leaves; *n* = 44), reproductive organs (flowers, stigma, fruit and seed; *n* = 12) and underground organs (rhizome, bulbs, root, tuber; *n* = 7). Latex and sap were mentioned for a single species, *Chelidonium majus* L. and *Opuntia ficus-indica* (L.) Mill., respectively. We note that for some species (*n* = 14), multiple organs were used.

#### 2.3.1. Local Therapeutic Categories

**Figure 3:**
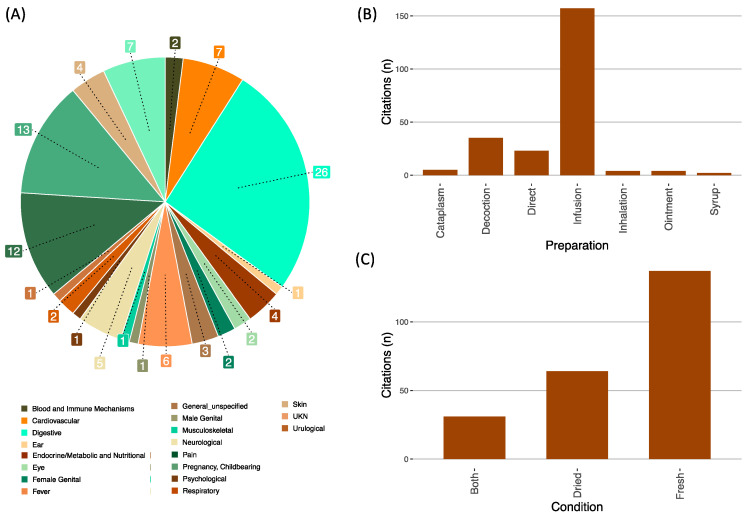
Details about human health afflictions treated with local plants. (**A**) Health disorders reported as categorized according to ICPC2. (**B**) Preparation mode of remedies. (**C**) Plant condition (fresh or dried) when preparing the remedy. General unspecified—all ills; UNK—specific illness unidentified.

Plants were used to treat human diseases linked to 17 ICPC-2 categories. The three most frequent uses concern the digestive system (*n* = 26; ICF = 69%), skin (*n* = 13, ICF = 70%) and respiratory system (*n* = 12; ICF = 62%) (Figure 3A). Other references included “general unspecified” disorders (six species), meaning the plant was used as a panacea; in addition, informants attributed medicinal interest for four species, yet could not recall the specific disease treated (labeled as “UNK”; Figure 3A). A remarkably high percentage of plants (74%) were used as multi-contextual remedies for several disorders (2–5 categories); only five species had a single medicinal purpose. The synergistic uses of plants for therapeutic purposes were rarely mentioned. By applying the same ICP2 categories to animal medicine, we found four categories of afflictions for which plants were used: “digestive” was the most mentioned with 11 species indicated; “urological”, “musculoskeletal” and “skin” were treated with one species each.

#### 2.3.2. Mode of Preparation and Administration in Human Medicine

The methods reported for internal use were infusion, decoction and syrup. The methods documented for external use included decoction (for washes and bath sitz), cataplasm, ointment and squeezing for the direct application of fresh material. Overall, the preferred form of using the species as medication was infusion (157 citations for thirty-three species; Figure 3B), followed by decoction (35 mentions for sixteen species; Figure 3B), and direct use (22 references for four species; Figure 3B). While the decoctions consisted of a heavy extraction in rolling boil water for harder plant parts (rhizome, whole plant), the infusions were used with softer parts (leaves, flowers, stigma) left to steep in very hot (not boiling) water. There were also reported instances of inhalations (respiratory; *Eucalyptus globus* Labill. and *Allium cepa* L.), cataplasms (e.g., *Plantago major* L.) and ointments (e.g., *Umbilicus rupestris* Salisb.), both applied for skin problems. Several species were used in different preparations; four species had three different modes of preparing the medicine pending the disease to be treated. The plants with medicinal purposes were harvested for immediate use (fresh; 27 species with 128 citations; Figure 3C), solely dried for preservation to use later (10 species mentioned 72 times; Figure 3C) or used in both conditions (32 references for 16 species; Figure 3C); for instance, *C. nobile*, *P. tridentatum*, *Plantago coronopus* L. and *Sambucus nigra* L. were used either fresh or dried; *A. cepa*, *E. globulus* and *U. rupestris* were used exclusively fresh; and *Hypericum androsaemum* L., *Hypericum perforatum* L. and *Calluna vulgaris* (L.) Hull were dried for later use. 

### 2.4. Association of Use Values (Ethnobotanical Indices) with Plant’s Ecological Features

Our results show that the number of uses, citation frequency, cultural importance, or number of vernacular names is not determined by plant origin, life form, or habitat of harvesting (Table 2). The same results held for the number of medicinal uses (MU) (Table 2). Overall, this suggests the studied communities harvest ethnobotanical resources regardless of the life forms and exploit all types of habitats.

## 3. Discussion

Integrative research exploring the relationship between biological and cultural diversity can provide solid ground for developing conservation strategies for biocultural diversity. Our study reveals, for the first time, the many fascinating uses of plants in MSES of NW Portugal. Ethnobotanical resources were multifunctional, i.e., the same species with different purposes and distinct parts of the plant with different uses, which support the idea of a large biocultural diversity. In the absence of any major geographical barrier separating plant assemblages, the ethnobotanical heritage is transmitted between similar MSES, as indicated by the lack of difference in ethnobotanical richness across the studied geographical territories. The shared species utilitarian species (24 species) and the consistency in the cultural dominance of some species—*P. tridentatum*, *Z. mays*, *E. arborea* and *R. graveolens*—also denotes territorial continuity.

As expected, medicinal use was the most often cited category. However, we did not anticipate the relevance of plants for fuel, less so its strong cultural role for ritualistic and superstitious purposes (Figure 1A,B). The high frequency of citation and the high consensus among informants regarding plant species used for medicine, fuel and rituals indicate that knowledge about these botanical resources, with (i) medicinal proprieties relevant for treating common human health symptoms, (ii) best combustible proprieties for cooking and warming, and (iii) attributed religiousness and magic power, are crucial for the livelihoods in these MSESs.

With this study, we aspire to help preserve local knowledge in its entirety. As such, our approach was based on curiosity rather than judgment, which led us to inquire about rituals (religious, magic, or superstitions). We discovered that a relevant portion of plants (10% of use reports, 6% of species) was used for religious and magical/sorcery purposes, with the most cited species being *Ruta graveolens*. The use of rue as a human and animal protector against misfortune and “evil eye” is still a current practice in the studied communities and is kept handy at home gardens, along with *Salvia rosmarinus* Spenn. and *Salvia officinalis* L. The ritualistic use of plants for religious or pagan ceremonies/beliefs is an important body of knowledge that should not be overlooked in studies aimed at the protection of biocultural heritage.

### 3.1. Biocultural Heritage—Biodiversity and Cultural Preservation

It is of utmost importance, and a worldwide quest, to document biocultural diversity before its extinction [23]. An essential portion of the biocultural heritage is the knowledge and practices associated with the use of wild plant species for various purposes. At the brink of a biological and cultural diversity crisis, ethnobotany is a topic of increasing interest Refs. [9,24]. Ethnobotany can simultaneously support the preservation of biocultural heritage and shrinking rural communities and contribute to biodiversity conservation in SCI-Rede Natura 2000 areas by supporting policies to protect plants (e.g., Refs. [25,26]) and the local knowledge inheriting/transmission system (e.g., Ref. [27]). In rural subsistence communities, local knowledge is pivotal for supporting the ecosystems’ regenerative capacity, which in turn provides a variety of services, including fuel, medicine and food. The prevalence of wild phanerophytes (trees and shrubs; 43%) and hemicryptophytes (perennials; 38%) as utilitarian plants translates the ecological knowledge about the ability of these life forms to cope with the harsh climatic conditions of higher elevations with increasing precipitation and decreasing temperature. The local reliance on predictable resources, for fulfilling the everyday needs regarding cultural and practical purposes, is consistent with altitudinal gradients in Southern Europe, where phanerophytes outcompete other plant life forms under mild conditions of intermediate elevations, causing hemicryptophytes to assume the dominant role in higher elevations [28,29,30]. With nearly 70% of the botanical resources collected in the wild and many (35 species) harvested in woodlands (oak and riparian forests), the impacts of global change on ethnobotanical diversity in mountain regions of NW Iberia should be a concern. These territories are also vulnerable to climate change, which may affect plant metabolomes and increase the risk of species extinction [31], and ultimately impact local ethnobotanical richness.

### 3.2. Ethnomedicine in Southern European Countries

In the Iberian and Italian peninsulas, the main focus of ethnobotany has been traditional medicine (e.g., Portugal [19,20,21,32]; Spain [33,34,35,36] and Italy [15,16]. Therefore, we here place our results in the perspective of other studies developed in Southern European countries. Ethnomedicine richness in the studied communities (53 species) was smaller in comparison to other Portuguese rural mountain communities: NE (88 species [20]), central (124 [32]), SW (105 [21]) and SE (150 [19]). Despite the lower richness, there was a good consensus regarding the use of medicinal plants (ICF = 0.78) when compared to ICF in other published studies in the Iberian Peninsula: Portugal (0.90 [21]; 0.85 [19]; 0.48 [20]), Spain (0.65 [34]; 0.71 [37]; 0.86 [36]). The plants used to treat stomach- and skin-related ailments had the highest agreement among the participants (ICF ~ 70%), indicating that these plants are well known in the community and further suggest their possible efficacy for these specific uses. The most frequently treated human health symptoms coincide with results obtained in works carried out in Iberia [19,21,35,38,39]. Likewise, in NW Portugal, plants in families Lamiaceae and Asteracea were the most employed to treat human afflictions. Our results provide basic and important data for further research aimed at pharmacological studies, and, especially, the ICF estimates can be useful for prioritizing plants for further pharmacological studies [40]. In fact, we are already exploring some of the plants documented.

### 3.3. Study Caveats

The current socio-demographic context of the communities (shrinking and aging) posed some challenges: the engagement of the informants in the sample is biased towards elders. Regarding this skew, the elderly population is expected to be the custodians of traditional knowledge, so the a priori bias towards elders was inescapable. However, to overcome that, we interviewed locals with <65 years (30% of informants). Pertaining the engagement, despite our effort to build a relationship of trust with constant visits to the community, some people were still very reluctant to spend time sharing information with us—allegedly busy with their affairs. We also suspect, from our conversations with people, that the ridicule of local knowledge has played a role in refraining some people from even discussing it. Our study was based on all plants reported, including the species only reported by one or two independent informants. In general, uses cited by least three independent informants is the threshold applied (e.g., Refs. [21,32,35]) to ensure reliability for future pharmacological prospects. Because we set out to document and analyze local knowledge and practices, we decided to report single and dual reports, as these may demonstrate the process of knowledge erosion.

### 3.4. Halting Local Knowledge Erosion

Several lines of evidence point to the idea of local knowledge erosion: the relatively low number of utilitarian species documented (*n* = 98 species), the ethnobotanical multifunctionality reported but mostly no longer in practice, the linguistic richness relatively lower (1.50) to other mountainous localities in the Iberian Peninsula (2.90 in Serra de Montejunto [21]; 1.72 in Serra de Montesinho [21,38]; 1.94 in l’Alt Empordà [33]) and the depopulation and aging of the MSES located at the southern edge of the Atlantic bioregion. Although we have no quantitative assessment of the loss, due to the lack of previous studies, to perform an explicit comparative analysis, all these elements, together with informant comments such as “you are 20 years late… the wisdom and actual users are already gone”, suggests that the wealth of local knowledge and practices failed to be transmitted over the last generations. The key to halting erosion will be to build awareness, especially among the younger generations, of the importance of this intangible cultural heritage and its safeguarding. There is a need to communicate results from studies like this. Besides the evident relevance of ethnomedicine through the search for phytochemicals with therapeutic proprieties, local knowledge and practices associated with botanical resources can be a source of information for (i) policy stakeholders to implement strategies for preservation of precious local knowledge that risks being lost due to modern standards, (ii) conservation authorities to ensure the sustainable use plant resources, (iii) other scientific endeavors and (iv) the economic sector to develop activities (gastronomy, eco-tourism) that can sustain local communities. Ultimately, this can revitalize the human–nature nexus and help preserve the local biocultural heritage in Alto Minho.

## 4. Materials and Methods

### 4.1. Characterization of the Study Area and Communities

**Figure 4:**
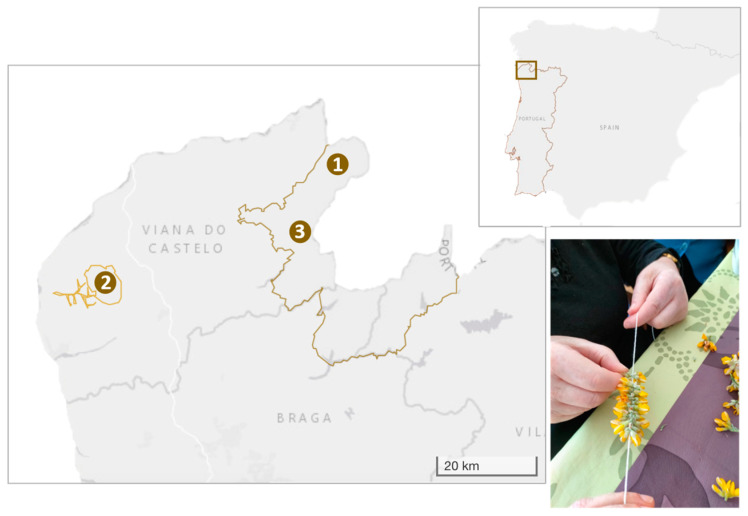
Geographic location of the three studied communities in NW Portugal: 1. Castro Laboreiro, 2. Serra d’Arga, 3. Soajo. Inset shows placement in the Iberian Peninsula. Photography of one informant teaching us how to use *Pterospartum tridentatum* flowers to make adornments.

Our study was conducted in three rural communities located in the mountains of Alto Minho region, NW Portugal (Castro Laboreiro, Serra d’Arga and Soajo; Figure 4). Alto Minho region hosts an incredibly rich cultural, linguistic and biological diversity; nearly 40% of this territory belongs to the Natura 2000 Network and the National Network of Protected Areas (RNAP). The study communities were selected according to four major criteria: (i) rural communities highly dependent on biological resources, i.e., socio-ecological systems, (ii) embedded in areas relevant for biological conservation, such as Special Areas of Conservation (SAC) in Natura 2000 network, with a wide topographical range which increases habitat diversity and hence plant richness (Table 3); (iii) peripheral territories, where the lack of opportunities and resources may facilitate the possibility of harboring deep-rooted customs and local peculiarities lessening the impacts of globalization; and (iv) strong demographic regression with high aging index, where, in the last decade, communities lost > 20% of the population and there are 10 times more elders than young people, contributing to a high risk of transmission break (Table 3).

### 4.2. Data Collection—Ethnobotanical Survey

We interviewed locals, both natural residents or long-time members of the community, by applying a snowball sampling approach, i.e., asking the informants to suggest further people with experience in traditional plant use [41]. Interviews were conducted between June 2022 and July 2023 using both semi-structured methods. Semi-structured interviews involved open-ended questions prepared in advance, allowing the interviewee to provide spontaneous and detailed responses. The questionnaire consisted of two parts: the first centered on the socio-demographic characteristics of the participant (age, educational level, profession/occupation and place of residence), and the second focused on the plant species used by the local community. For this second part, we collected vernacular names, folk uses, plant parts used (e.g., root, shoot, leaves, flowers), plant condition when used (fresh, dried, both), time of collection (month/season) and habitat of harvest (8 categories). When reported for medicinal purposes, the informants were also asked to identify the diseases the plant treated, the remedy preparation (e.g., infusion, decoction, cataplasm) and application method (e.g., internal, external).

Due to the socio-demographic context of the communities most informants were elderly and many with mobility limitations for outings in the field to identify plants. To circumvent this issue, we adapted our approach and opted for a visual identification either of plants collected by us in the field or through photographs in guides and databases, namely Flora-on (https://flora-on.pt/ accessed 2022–2023) from the Portuguese Botanical Society. In cases of ambiguity (several), we discarded the reference.

Data were recorded using two methods: on paper and audio recordings, mostly simultaneously. All information was registered, transcribed and introduced into a database. As a general procedure in ethnobotanical surveys, the information was organized in use reports and use categories (Table 4): human medicinal, veterinary medicinal, human food, animal food, rituals, cosmetics/hygiene, utensils, ornamental, gastronomy, aromatic, fuel, dyes, clothing, and others (for uses that did not fit into any of the previous). For medicinal uses, we categorized the diseases treated with medicinal plants according to the International Classification of Primary Care (ICPC) 2nd edition, which is accepted by the United Nations’ World Health Organization (WHO). The ICPC2 categories are as follows: blood, blood-forming organs and immune mechanisms; digestive; eye; ear; cardiovascular; musculoskeletal; neurological; psychological; respiratory; skin; endocrine/metabolic and nutritional; urological; pregnancy, childbearing, family planning; female genital; and male genital. When multiple and unspecified diseases were mentioned, we grouped those into “global unspecified”. In a few cases, the informant knew that the plant had medicinal uses, yet could not recall which, hence we created the category “unknown”.

### 4.3. Botanical Identification, Ecology and Conservation Status

Plant nomenclature followed the Flora Iberica [42], Nova Flora de Portugal [43]. Voucher specimens were prepared and deposited at the Herbarium of the Natural History and Science Museum of the University of Porto (PO; Portugal; numbers PO-V72999 to PO-V73023; Supplementary Materials Table S1) and scientific names and families were verified. We categorized the mentioned plants according to life form sensu Raunkiær (1934), which classifies plants according to the place where the growth point is located during the less favorable season: phanerophyte (growth point projects into the air, e.g., trees and bushes), chamaephyte (growth point at or near the soil surface; many perennial herbaceous plants), hemicryptophyte (growth point at the ground level protected by withered leaves and soil), geophytes (growth point below ground level; tuber, rhizome, bulbs), therophyte (survive unfavorable period as seeds) and epiphyte (grow on or is attached to other living plants). In addition, we also collected information regarding its origin (native vs. exotic) and the conservation status in Portugal according to the current Red List [44].

### 4.4. Quantitative Ethnobotanical Indexes

To summarize the ethnobotanical information collected and later compare it with similar studies, we estimated the indices described below. But first, we converted the data into a “use report” (UR), where each UR corresponds to an event where an informant reported a utilitarian species to a particular category of use. The number of useful botanical species was estimated as Ethnobotanical Richness (R [45]). The Relative Frequency Citation (RFC [46]) was calculated by dividing the number of informants who claim to have used/used the plant species (*FC*) by the total number of informants in the survey (*N*). The formula is RFC = *FC*/*N*, with *FC* (frequency of citation) being the total number of informants that referred to the taxon and *N* being the total number of informants in the survey. The cultural importance index (CI [46]) is used to evaluate the extent to which each species is present in the local culture and the memory of the informants. The index is estimated as CI = *UR*/N, where *UR* is the number of different uses mentioned for each taxon and *N* is the total number of informants interviewed during the ethnobotanical survey.

To determine whether there is agreement among respondents in the use of plant species in each category, we estimated the informant consensus factor (ICF [47]). ICF was calculated as (*Nur* − *Nt*)/(*Nur* − 1), where Nur is the number of use reports in each use category and Nt is the number of species used in the same category by all informants. ICF values range from 0 to 1, and the larger the index, the better the agreement among respondents, and hence an indication of well-established criteria for the use of plant species.

The names of plants, phytonyms, are an important proxy of cultural heritage and an indicator of the erosion of traditional knowledge about biodiversity. Hence, the diversity in phytonym was assessed using (i) linguistic diversity index [33], which is the mean number of folk names for the plants recorded during the study, and (ii) botanical linguistic richness (BLR), which is calculated as the ratio between the number of vernacular names and the number of species scientific name. BLR > 1 indicates that taxonomic entities have multiple vernacular designations, which translates the linguistic richness within the community.

### 4.5. Statistic Analyses: Sampling Assessment and Ecological Effects on Ethnobotanical Information

To assess the level of plant documentation, i.e., adequate sampling, we used accumulation curves based on the number of citations associated with each plant. The accumulation curve with 95% confidence intervals suggests that our inventory is nearly complete. We reached 98 species with 44 informants; however, if we were to continue with fieldwork, it would be likely that we have added a few other reports of less used plants (bootstrap mean number of species = 109, SE = 4; Supplementary Materials Figure S2). Therefore, given the absence of earlier reports and the socio-demographic context (see Section 4.1), we contend that our data allows for the first grasp of the ethnobotany of the studied communities.

Before formal tests, we assessed data normality using quantile–quantile plots and Tukey’s test. Because variables were non-normal, we used non-parametric Kruskal–Wallis tests to evaluate whether (i) the number of plant citations varied significantly with locality and (ii) to compare use values among ecological features of plants. Specifically, we tested whether there was an association between plant life form, origin (wild vs. cultivated), habitat of collection and plant condition when used (fresh or dried) with the number of citations, relative frequency of citation, cultural importance, number of medicinal uses and number of vernacular names. We report χ^2^ values and *p*-values < 0.05 were considered statistically significant. All statistical analyses were performed using R (version 4.2.3) and R Studio (version 2022.02.3+492) with packages vegan, ggplot2 and ggstatsplot.

### 4.6. Ethical Considerations

While conducting the questionnaires, we adhered to ethical principles as follows: (i) full disclosure—the informants were fully acquainted with the scope and goal of the research; (ii) prior informed voluntary consent—all informants agreed to the terms of the research and provided oral and/or written consent; (iii) and confidentiality—we ensured the anonymity and privacy of the respondents. The study was conducted following the Declaration of Helsinki and approved by the Ethics Committee for Social, Life and Health Sciences of the Polytechnic Institute of Viana do Castelo (CECSVS2023/09/ii). In addition, we observed the International Society of Ethnobiology Code of Ethics. The plant vouchers were collected considering the permit issued by the national authority (ICNF, Licenses REC546-2022 and REC29-2023). 

## Figures and Tables

**Table 1:** Informant consensus factor (ICF). The factor was calculated (Nur − Nt)/(Nur − 1). Values range from 0 to 1, and the larger the factor, the better the agreement among informants.

Category	Nur	Nt	ICF
Aromatic	7	4	0.50
Clothing	12	8	0.36
Fuel	59	13	0.79
Cosmetic/Hygiene	12	5	0.64
Dye	2	2	0.00
Food Animal	30	13	0.59
Food Human	31	15	0.53
Gastronomy	37	14	0.64
Medicinal Human	232	52	0.77
Medicinal Veterinary	23	14	0.41
Ornamental	8	5	0.43
Other	76	28	0.64
Ritual	63	12	0.82
Utensil	44	15	0.67

**Table 2:** Summary of the Chi-squared tests (*χ*^2^) of independence for number of uses (NU), relative frequency of citation (RFC), cultural importance (CI), number of vernacular names (VN) and number of medicinal uses (MU). Dependent and explanatory variables are numeric and categorial, respectively.

	NU	RFCs	CI	VN	MU
**Origin** (2 categories)	*χ*^2^ = 5.79	*χ*^2^ = 32.59	*χ*^2^ = 44.14	*χ*^2^ = 7.31	*χ*^2^ = 13.61
	*p* = 0.93	*p* = 0.44	*p* = 0.38	*p* = 0.29	*p* = 0.19
	V_Cramer_ = 0.00	VC_ramer_ = 0.04	VC_ramer_ = 0.09	V_Cramer_ = 0.08	V_Cramer_ = 0.18
**Life form** (6 categories)	*χ*^2^ = 25.28	*χ*^2^ = 83.22	*χ*^2^ = 117.24	*χ*^2^ = 8.97	*χ*^2^ = 13.10
	*p* = 0.71	*p* = 0.38	*p* = 0.20	*p* = 0.88	*p* = 0.87
	V_Cramer_ = 0.00	V_Cramer_ = 0.07	V_Cramer_ = 0.15	V_Cramer_ = 0.00	V_Cramer_ = 0.00
**Habitat** (8 categories)	*χ*^2^ = 36.38	*χ*^2^ = 110.58	*χ*^2^ = 139.60	*χ*^2^ = 27.71	*χ*^2^ = 22.73
	*p* = 0.72	*p* = 0.52	*p* = 0.66	*p* = 0.15	*p* = 0.83
	V_Cramer_ = 0.00	V_Cramer_ = 0.00	V_Cramer_ = 0.00	V_Cramer_ = 0.15	V_Cramer_ = 0.00

V_Cramer_ [0.10–0.20] indicates a weak association.

**Table 3:** Relevant ecological and social features: conservation, plant biodiversity, altitude and demographic trends in the studied communities.

	Castro Laboreiro	Serra d’Arga	Soajo
**Conservation interest**	SAC—PTCON0001 Peneda-Gerês National Park Gerês-Xurés Biosphere Reserve	SAC—PTCON0039	SAC—PTCON0001 Peneda-Gerês National Park Gerês-Xurés Biosphere Reserve
**Vascular flora (nb species)**	817	546	817
**Topographical range (m)**	600–1300	250–800	200–1400
**Demographic trend 2011–2021 § (%)**	−23.4%	−23%	−32%
**Aging Index ¥**	2079	1800	989

estimation for the Peneda-Gerês National Park; § INE—statistics Portugal, CENSOS 2021; ¥ AI = (n ind. ≥ 65 yo/ n ind. (0–14 yo) × 100).

**Table 4:** Use categories (14) considered in this study when collecting field data.

Use Category	Description
Human Medicinal	medicinal proprieties for humans
Veterinary Medicinal	medicinal proprieties for livestock
Human Food	direct human consumption
Animal Food	livestock feeding
Rituals	religious ceremonies and magical purposes
Gastronomy	transformed by cultural practices for human consumption
Cosmetics/Hygiene	body care and beauty products
Utensils	manufacture household and agricultural utensils
Ornamental	adornment of objects, houses and patio
Aromatic	food flavoring and condiment
Fuel	heating, cooking and smoking/curing food
Dyes	textile coloring
Clothing	manufacture of clothes
Others	purposes not fitting any of other use category

## Data Availability

We have included all data related to this study either in the main text or as Supplementary Materials.

## Supplementary Materials

The following supporting information can be downloaded at https://www.mdpi.com/article/10.3390/plants13192824/s1, Table S1: Details about the 98 plant species reported. Figure S1: Number of shared and unique plants species documented in the three study communities. Figure S2: Accumulation curve based on number of species documented as new informants were incorporated in the study.
