# Supplementary material for: Ethnobotanical Inventory of Plants Used by Mountainous Rural Communities in NW Portugal
Source: Plants (Basel). 2024 Oct 9;13(19):2824. doi: 10.3390/plants13192824 (PMC11479140; doi:10.3390/plants13192824)
Supplement: Supplementary file 1 [file plants-13-02824-s001.zip › SuppMaterial_FigS1-S2.pdf]

Supplementary Material, Figures S1 and S2, for "Ethnobotanical Inventory of Plants Used by Mountainous Rural Communities in NW Portugal" by Sá et al. 2024.

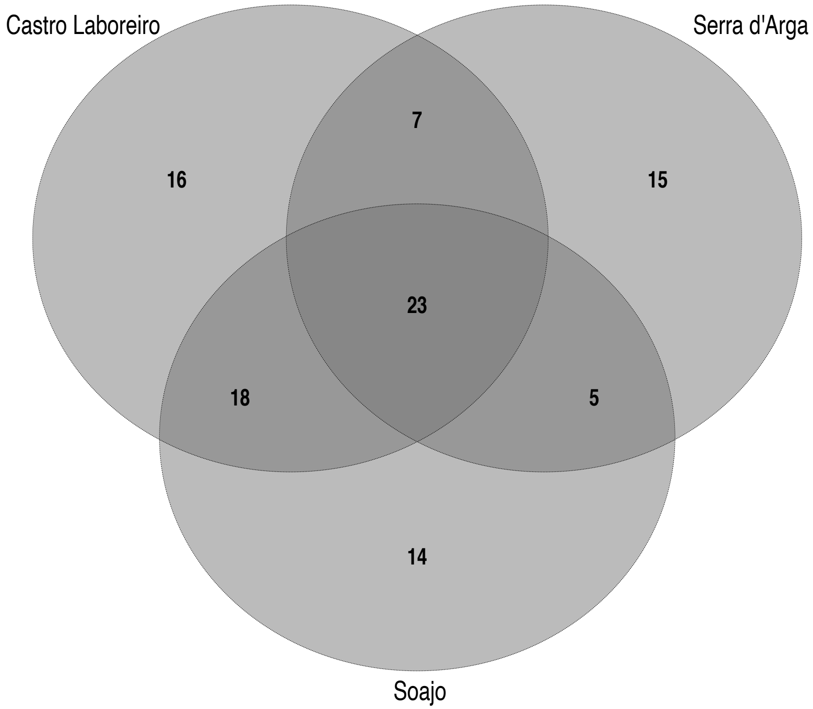

Figure S1: Number of shared and unique plants species documented in the three study communities. Twenty-three species are share among among the three.

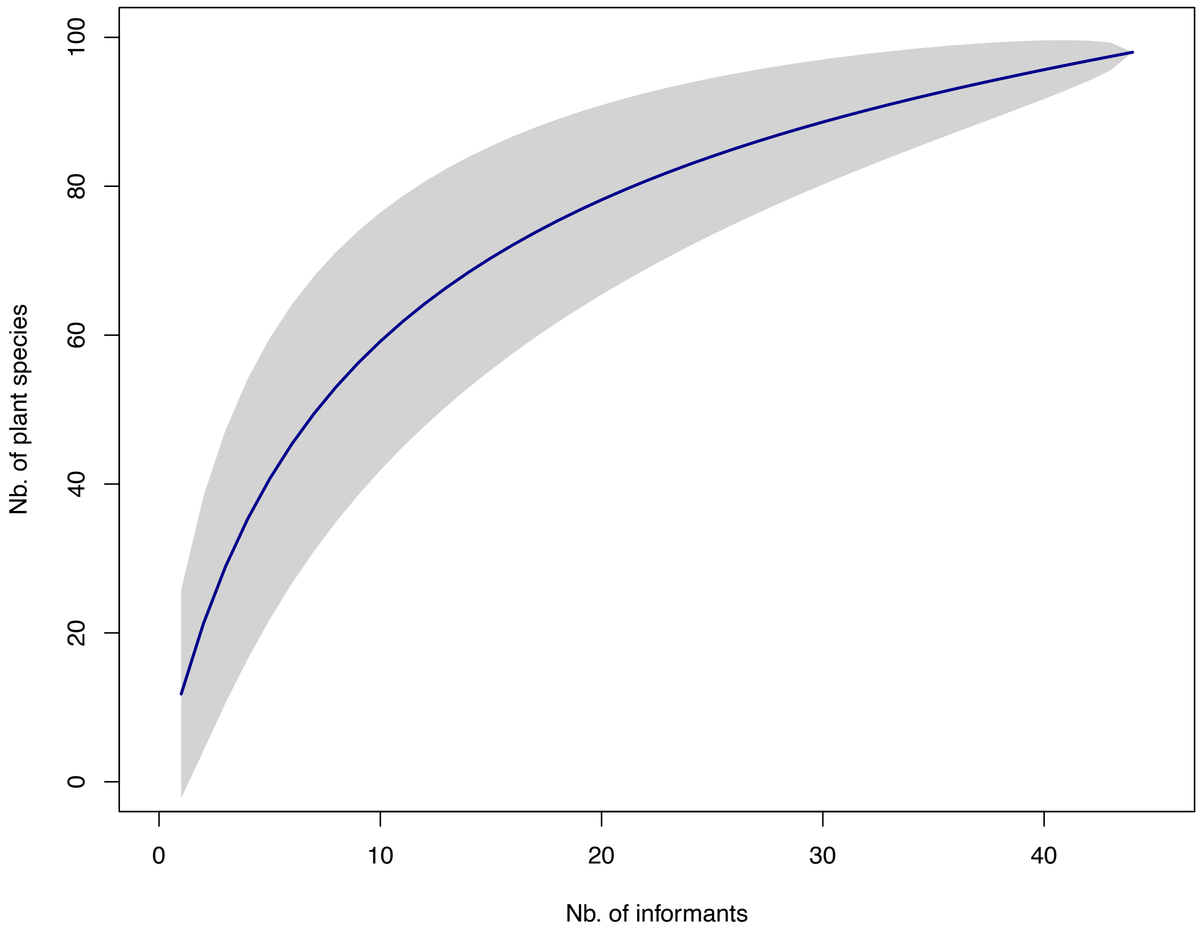

Figure S2: Accumulation curve based on number of species documented as new informants were incorporated in the study. The extrapolated total number of plant species was estimated using the function "specpool" from the "vegan" R package.
