# Supplementary material for: Ethnobotanical Inventory of Plants Used by Mountainous Rural Communities in NW Portugal
Source: Plants (Basel). 2024 Oct 9;13(19):2824. doi: 10.3390/plants13192824 (PMC11479140; doi:10.3390/plants13192824)
Supplement: Supplementary file 1 [file plants-13-02824-s001.zip › SuppMaterial_SuppTable1.pdf]

Table S1. Details about the 98 plant species reported. Botanical family, species scientific name conservation status in Portugal, origin, habitat of harvest, life form *sensu* Raunkiær, use reports (UR), number of uses (NU), relative frequency of citation (RFC), cultural importance (CI) and number of vernacular names (VN).

Family	Species name (voucher nb)	Conservation	Origin	Habitat	Life Form	UR	Nus	RFCs	CI	VN
Amaryllidaceae	*Allium cepa* L.	None	Cultivated	Agricultural field	Geophyte	5	2	0.114	0.114	1
Amaryllidaceae	*Allium sativum* L.	None	Cultivated	Agricultural field	Geophyte	6	2	0.114	0.136	1
Apiaceae	*Apium graveolens* L.	None	Cultivated	Home garden	Hemicryptophyte	3	1	0.068	0.068	1
Apiaceae	*Daucus carota* L.	None	Cultivated	Agricultural field	Hemicryptophyte	1	1	0.023	0.023	1
Apiaceae	*Foeniculum vulgare* Mill.	None	Wild	Fallow field	Hemicryptophyte	12	4	0.182	0.273	2
Aquifoliaceae	*Ilex aquifolium* L.	Least Concern	Wild	Woodland	Phanerophyte	4	4	0.068	0.091	1
Asparagaceae	*Polygonatum odoratum* (Mill.) Druce (PO-V73013-14)	None	Wild	Meadow	Geophyte	4	1	0.091	0.091	1
Aspleniaceae	*Asplenium trichomanes* L. (PO-V73012)	None	Wild	Rupicolous	Hemicryptophyte	8	1	0.182	0.182	3
Asteraceae	*Achillea millefolium* L.	None	Wild	Fallow field	Chamaephyte	7	5	0.091	0.159	3
Asteraceae	*Artemisia absynthus* L.	None	Cultivated	Home garden	Hemicryptophyte	4	2	0.068	0.091	1
Asteraceae	*Chamaemelum nobile* (L.) All. (PO-V73002)	None	Wild	Meadow	Hemicryptophyte	17	2	0.318	0.386	3
Betulaceae	*Alnus glutinosa* (L.) Gaertn	None	Wild	Riparian	Phanerophyte	3	2	0.068	0.068	1
Betulaceae	*Betula pubescens* Ehrh.	None	Wild	Riparian	Phanerophyte	10	4	0.182	0.227	3
Betulaceae	*Corylus avellana* L.	None	Wild	Woodland	Phanerophyte	1	1	0.023	0.023	1
Boraginaceae	*Lithodora prostrata* (Loisel.) Griseb.	None	Wild	Scrubland	Chamaephyte	2	2	0.045	0.045	1
Buxaceae	*Buxus sempervivens* L.	Endangered	Cultivated	Home garden	Phanerophyte	1	1	0.023	0.023	1
Cactaceae	*Opuntia ficus-indica* (L.) Miller.	Exotic	Cultivated	Home garden	Phanerophyte	1	1	0.023	0.023	1
Caprifoliaceae	*Lonicera periclymenum* L.	None	Wild	Woodland	Phanerophyte	1	1	0.023	0.023	1
Caprifoliaceae	*Sambucus nigra* L. (PO-V73018)	None	Wild	Riparian	Phanerophyte	14	3	0.273	0.318	3
Cistaceae	*Cistus psilosepalus* Sweet	Least Concern	Wild	Scrubland	Phanerophyte	5	2	0.114	0.114	2
Crassulaceae	*Sedum brevifolium* DC.	None	Wild	Rupicolous	Hemicryptophyte	2	1	0.045	0.045	2
Crassulaceae	*Umbilicus rupestris (Salisb.) Dandy* (PO-V73022)	None	Wild	Rupicolous	Hemicryptophyte	10	3	0.205	0.227	3
Cytinaceae	*Cytinus hypocistis* (L.) L.	None	Wild	Scrubland	Epiphyte	2	1	0.045	0.045	1

Dennstaedtiaceae	*Pteridium aquilinium* (L.) Kuhn	None	Wild	Fallow field	Phanerophyte	1	1	0.023	0.023	1
Ericaceae	*Arbutus unedo* L.	None	Wild	Scrubland	Phanerophyte	5	2	0.091	0.114	2
Ericaceae	*Calluna vulgaris* (L.) Hull	None	Wild	Scrubland	Phanerophyte	11	4	0.182	0.250	5
Ericaceae	*Daboecia cantabrica* (Huds.) K. Koch	None	Wild	Scrubland	Phanerophyte	1	1	0.023	0.023	1
Ericaceae	*Erica arborea* L.<br>(PO-V73003)	None	Wild	Scrubland	Phanerophyte	5	4	0.409	0.591	3
Ericaceae	*Erica cinerea* L.	None	Wild	Scrubland	Phanerophyte	3	1	0.114	0.114	3
Ericaceae	*Erica umbellate* L .	None	Wild	Scrubland	Phanerophyte	5	1	0.068	0.068	2
Ericaceae	*Vaccinium myrtillus* L.<br>(PO-V73023)	None	Wild	Woodland	Phanerophyte	4	2	0.091	0.091	1
Fabaceae	*Acacia longifolia* (Andrews) Willd.	Exotic	Wild	Fallow field	Phanerophyte	1	1	0.023	0.023	1
Fabaceae	*Cytisus multiflorus* (L'Hér.) Sweet	None	Wild	Scrubland	Phanerophyte	1	1	0.023	0.023	1
Fabaceae	*Cytisus scoparius* (L.) Link	None	Wild	Scrubland	Phanerophyte	6	3	0.114	0.409	2
Fabaceae	*Cytisus striatus* (Hill)Rothm.	None	Wild	Scrubland	Phanerophyte	18	3	0.318	0.227	3
Fabaceae	*Lupinus albus* L.	None	Cultivated	Agricultural field	Therophyte	3	2	0.068	0.068	1
Fabaceae	*Pterospartum tridentatum* (L.) Link<br>(PO-V73015-17)	None	Wild	Scrubland	Phanerophyte	46	7	0.545	1.045	1
Fabaceae	*Ulex europaeus* L.	None	Wild	Scrubland	Phanerophyte	6	2	0.114	0.136	2
Fabaceae	*Ulex minor* Roth	None	Wild	Scrubland	Phanerophyte	15	3	0.273	0.341	2
Fagaceae	*Castanea sativa* Mill.	None	Wild	Woodland	Phanerophyte	1	1	0.023	0.023	1
Fagaceae	*Quercus pyrenaica* Willd.	None	Wild	Woodland	Phanerophyte	10	4	0.159	0.227	2
Fagaceae	*Quercus robur* L.	None	Wild	Woodland	Phanerophyte	17	4	0.295	0.386	2
Fagaceae	*Quercus suber* L.	None	Wild	Woodland	Phanerophyte	6	3	0.114	0.136	1
Geraniaceae	*Geranium robertianum* L.<br>(PO-V73004)	None	Wild	Fallow field	Therophyte	3	1	0.068	0.068	1
Hypericaceae	*Hypericum androsaemum* L.	None	Both	Home garden	Chamaephyte	7	1	0.159	0.159	1
Hypericaceae	*Hypericum perforatum* L.<br>(PO-V73005)	None	Wild	Scrubland	Chamaephyte	2	1	0.045	0.045	2
Juglandaceae	*Juglans regia* L.	None	Cultivated	Agricultural field	Phanerophyte	1	1	0.023	0.023	1
Juncaceae	*Juncus effusus L.*	None	Wild	Meadow	Hemicryptophyte	1	1	0.023	0.023	1
Lamiaceae	*Calamintha nepeta* (L.) Savi<br>(PO-V73001)	None	Cultivated	Home garden	Hemicryptophyte	4	3	0.068	0.091	1
Lamiaceae	*Lavandula stoechas* L.	None	Wild	Scrubland	Phanerophyte	1	1	0.023	0.023	1

Lamiaceae	*Melissa officinalis* L. (PO-V73007)	None	Cultivated	Home garden	Hemicryptophyte	7	1	0.159	0.159	1
Lamiaceae	*Melittis melissophyllum* L. (PO-V73008)	None	Wild	Woodland	Hemicryptophyte	15	2	0.318	0.341	1
Lamiaceae	*Mentha x gentilis* L. (PO-V73009)	None	Cultivated	Home garden	Hemicryptophyte	2	2	0.023	0.045	1
Lamiaceae	*Mentha x piperita* L.	None	Cultivated	Home garden	Hemicryptophyte	2	1	0.045	0.045	2
Lamiaceae	*Mentha aquatica* L. (PO-V73010)	None	Cultivated	Home garden	Hemicryptophyte	3	1	0.068	0.068	1
Lamiaceae	*Mentha pulegium* L.	None	Cultivated	Home garden	Hemicryptophyte	3	2	0.068	0.068	1
Lamiaceae	*Mentha suaveolens* Ehrh. (PO-V73011)	None	Wild	Fallow field	Hemicryptophyte	6	4	0.091	0.136	1
Lamiaceae	*Origanum vulgare* (Hoffmanns & Link) Bonnier & Layens	None	Wild	Scrubland	Chamaephyte	3	3	0.045	0.068	2
Lamiaceae	*Salvia microphylla* Kunth	Exotic	Cultivated	Home garden	Hemicryptophyte	2	1	0.045	0.045	2
Lamiaceae	*Salvia officinalis* L.	None	Cultivated	Home garden	Hemicryptophyte	15	4	0.273	0.341	2
Lamiaceae	*Salvia Rosmarinus* (L.) Scheid.	None	Cultivated	Home garden	Phanerophyte	18	5	0.318	0.409	1
Lamiaceae	*Thymus caespititius* Brot. (PO-V73020-21)	None	Wild	Scrubland	Hemicryptophyte	8	3	0.182	0.182	1
Lamiaceae	*Thymus vulgaris* L.	None	Wild	Scrubland	Hemicryptophyte	1	1	0.023	0.023	1
Lauraceae	*Laurus nobilis* L.	None	Wild	Riparian	Phanerophyte	19	**9**	0.341	0.432	2
Liliaceae	*Asphodelus macrocarpus* Parl.	None	Wild	Woodland	Geophyte	1	1	0.023	0.023	1
Linaceae	*Linum usitatissimum* L.	None	Cultivated	Agricultural field	Hemicryptophyte	4	3	0.091	0.091	1
Malvaceae	*Malva sylvestris* L. (PO-V73006)	None	Wild	Fallow field	Hemicryptophyte	13	2	0.273	0.295	1
Myrtaceae	*Eucalyptus globulus* Labill.	Exotic	Wild	Woodland	Phanerophyte	1	2	0.136	0.136	3
Oleaceae	*Fraxinus angustifolia* Vahl	None	Wild	Riparian	Phanerophyte	2	1	0.045	0.045	1
Oleaceae	*Olea europaea* (Mill.) Lehr	None	Cultivated	Agricultural field	Phanerophyte	15	7	0.273	0.341	1
Osmundaceae	*Osmunda regalis* L.	None	Wild	Riparian	Hemicryptophyte	5	1	0.114	0.114	1
Papaveraceae	*Chelidonium majus* L.	None	Wild	Fallow field	Hemicryptophyte	7	2	0.159	0.159	1
Phytolaccaceae	*Phytolacca americana* L.	Exotic	Wild	Fallow field	Hemicryptophyte	1	1	0.023	0.023	1
Pinaceae	*Pinus pinaster* Aiton	None	Wild	Woodland	Phanerophyte	4	1	0.091	0.091	1
Plantaginaceae	*Digitalis purpurea* L.	None	Wild	Fallow field	Hemicryptophyte	26	1	0.023	0.023	1
Plantaginaceae	*Plantago coronopus* L.	None	Wild	Fallow field	Hemicryptophyte	3	1	0.068	0.068	2

	(PO-V73000)									
Plantaginaceae	*Plantago major* L.	None	Wild	Fallow field	Hemicryptophyte	2	1	0.045	0.045	2
Poaceae	*Secale cereale* L.	None	Cultivated	Agricultural field	Therophyte	32	4	0.341	0.727	1
Poaceae	*Triticum aestivum* L.	None	Cultivated	Agricultural field	Hemicryptophyte	2	2	0.023	0.045	1
Poaceae	*Zea mays* L.	None	Cultivated	Agricultural field	Therophyte	28	7	0.341	0.636	1
Polygonaceae	*Rumex acetosa* L.	None	Wild	Fallow field	Hemicryptophyte	1	1	0.023	0.023	1
Polygonaceae	*Rumex obtusifolius* L.	None	Wild	Fallow field	Hemicryptophyte	2	2	0.045	0.045	1
Polygonaceae	*Ruscus aculeatus* L.	None	Wild	Woodland	Geophyte	3	1	0.068	0.068	2
Portulacaceae	*Montia fontana* L.	None	Wild	Meadow	Hemicryptophyte	1	1	0.023	0.023	1
Primulaceae	*Primula acaulis* (L.) L.	None	Wild	Riparian	Hemicryptophyte	1	1	0.023	0.023	1
Rosaceae	*Fragaria vesca* L.	None	Wild	Woodland	Hemicryptophyte	3	2	0.068	0.068	2
Rosaceae	*Malus sylvestris* Mill.	None	Wild	Woodland	Phanerophyte	1	1	0.023	0.023	1
Rosaceae	*Prunus avium* L.	None	Cultivated	Home garden	Phanerophyte	3	3	0.068	0.068	1
Rutaceae	*Citrus limon* (L.) Burm. f.	None	Cultivated	Home garden	Phanerophyte	3	1	0.068	0.068	1
Rutaceae	*Citrus x sinensis* (L.) Osbeck	None	Cultivated	Home garden	Phanerophyte	11	2	0.250	0.250	1
Rutaceae	*Ruta graveolens* L.	None	Cultivated	Home garden	Hemicryptophyte	22	3	0.409	0.500	2
Salicaceae	*Populus nigra* L.	Exotic	Wild	Riparian	Phanerophyte	1	1	0.023	0.023	1
Salicaceae	*Salix atrocinerea* Brot.	None	Wild	Riparian	Phanerophyte	8	3	0.159	0.182	2
Saxifragaceae	*Saxifraga spathularis* Brot. (PO-V73019)	None	Wild	Rupicolous	Phanerophyte	3	1	0.068	0.068	2
Solanaceae	*Solanum tuberosum* L.	None	Cultivated	Agricultural field	Geophyte	3	3	0.045	0.068	1
Urticaceae	*Urtica dioica* L.	None	Wild	Fallow field	Hemicryptophyte	12	3	0.227	0.273	1
Verbenaceae	*Aloysia citrodora* Palàu (PO-V72999)	Exotic	Cultivated	Home garden	Phanerophyte	5	1	0.114	0.114	3
Violaceae	*Viola rivinianaI Rchb.*	None	Wild	Riparian	Hemicryptophyte	1	1	0.023	0.023	1

PO-V*x*: voucher number at the herbarium of MHNC-UP; further details in the main text, section methodology.
